# From Collaboration to Identity: The Role of Social Norms and Interdependence in Medical Students

**DOI:** 10.3390/bs16081320

**Published:** 2026-08-03

**Authors:** Elvira Rodriguez-Flores, Maria Valentina Toral-Murillo, Fernanda Medina-Vinelli, Karime Nereida Valdez-Toral, Karla Vanessa Gonzalez-Contreras, Yussef Esparza-Guerrero, Yasaman Gomez-Mazinani, Maricela Casas-Castañeda, Eli Efrain Gomez-Ramirez

**Affiliations:** 1 Unidad Académica de Medicina, Instituto de Ciencias Biológicas, Universidad Autónoma de Guadalajara, Zapopan 45129, Mexico; elvira.rodriguez@edu.uag.mx (E.R.-F.); valentina.toral@edu.uag.mx (M.V.T.-M.); yussef.esparza@edu.uag.mx (Y.E.-G.); mcasas@edu.uag.mx (M.C.-C.); 2 Programa de Servicio Social en Investigación, Facultad de Medicina, Instituto de Ciencias Biológicas, Universidad Autónoma de Guadalajara, Zapopan 45129, Mexico; fernanda.medina@edu.uag.mx (F.M.-V.); karime.valdez@edu.uag.mx (K.N.V.-T.); yasaman.gomez@edu.uag.mx (Y.G.-M.); 3 Programa de Semillero Científico, Facultad de Medicina, Instituto de Ciencias Biológicas, Universidad Autónoma de Guadalajara, Zapopan 45129, Mexico; kvanessa.gonzalez@edu.uag.mx; 4 Departamento de Fisiología, Instituto de Terapéutica Experimental y Clínica (INTEC), Universidad de Guadalajara, Guadalajara 44340, Mexico

**Keywords:** social norms, social cognition, social interdependence, cooperative behavior, professional identity

## Abstract

Social Interdependence Theory posits that cooperative behavior emerges when individuals perceive that their outcomes depend on the actions of others, a dynamic that is central to collaborative learning in medical education. Professional identity formation is recognized as a key developmental process during early medical training; however, quantitative evidence examining its association with collaborative interdependence in preclinical cohorts remains limited. This cross-sectional, correlational, non-experimental study examined the associations between perceived social interdependence and professional identity among 108 fourth-semester medical students enrolled in a Musculoskeletal and Integumentary System course. Professional identity was assessed using the Professional Identity Questionnaire, and social interdependence was measured using the Social Interdependence in Collaborative Learning Scale. To explore the relationship between these constructs, we conducted a series of descriptive and inferential statistical analyses. The results indicated generally high levels of professional attachment and a strong sense of belonging to the medical profession. Dimensions of social interdependence, particularly boundary interdependence and collaborative work, showed strong positive associations, while professional identity was also positively related to attachment. Regression analyses further suggested that collaborative work remained independently associated with professional identity after accounting for relevant sociodemographic and vocational factors. These findings indicate that positive interdependence—particularly group cohesion and shared boundaries—is associated with higher levels of professional identity. Strengthening collaborative learning structures in early medical education may support students’ sense of belonging, norm internalization, and commitment to the medical profession.

## 1. Introduction

Social norms and social cognition shape cooperation, particularly in social dilemmas where individuals balance personal and collective interests. This topic is especially relevant in medical education, particularly during the preclinical stage, where learning often occurs through collaborative activities. In these environments, students’ ability to manage interdependence and coordinate efforts toward shared goals is essential for academic success and effective curriculum design (Wittek et al., 2025).

According to Social Interdependence Theory (SIT), individuals’ outcomes depend not only on their own actions but also on those of other group members. In educational settings, the structure of goals and tasks shapes student interaction, and positive interdependence promotes cooperation by leading students to perceive that they succeed only if others succeed as well. This process is supported by three mechanisms described by Morton Deutsch (Johnson & Johnson, 2009). The development of professional identity and collaborative competencies is essential in early medical education, as both are closely linked to students’ learning experiences and future professional practice. Social interdependence plays a critical role in shaping these experiences, influencing how individuals interact, share responsibilities, and achieve common goals in academic settings. In this context, collaborative learning environments foster not only knowledge acquisition but also the internalization of professional values and norms. Furthermore, positive interdependence among students may strengthen their sense of belonging and commitment to the medical profession, thereby contributing to their professional identity formation.

SIT also identifies three forms of interdependence: outcome (shared goals), means (resources, roles, and tasks), and boundary (group identity and cohesion). Recent research in social cognition suggests that individuals also infer their level of interdependence during interactions (Johnson & Johnson, 2009). These inferences include perceptions of mutual dependence, power asymmetries, and alignment or conflict of interests, which influence decisions about cooperation, collective effort, and the sanctioning of opportunistic behaviors (free riders) (Balliet & Lindström, 2023; Colnaghi et al., 2025).

From the perspective of Social Identity Theory, part of an individual’s self-concept derives from belonging to social groups. When group identity becomes salient, people begin to see themselves not only as individuals but as members of a collective. In this context, group norms act as behavioral guides that indicate what actions are appropriate, such as “what a good team member does” (Wittek et al., 2025; Whelan, 2025). These norms function as group prototypes—models of typical or ideal behavior—that shape conformity and encourage cooperation according to the expectations of the ingroup, even without external supervision (Whelan, 2025).

In collaborative learning contexts, this perspective explains how structural interdependence gains behavioral meaning through shared norms and how a sense of belonging motivates members to contribute to collective goals (Whelan, 2025; Shimizu et al., 2020). In digital environments the formation of social identity and group norms may be mediated by platforms and algorithms, making group membership more flexible and dynamic across multiple contexts (Wittek et al., 2025).

Professional identity formation (PIF) is understood as a deep process of “being and becoming” that goes beyond acquiring knowledge or demonstrating professional behaviors. According to Sylvia Cruess and Yvonne Steinert, the ultimate goal of medical education is not only for students to act like physicians, but to become physicians (Verlind-Brouwer et al., 2025). This view expands George E. Miller’s Pyramid by adding the level of “Is” at the top, emphasizing that internalizing professional values and responsibilities represents the highest level of training. From this perspective, professionalism becomes an identity transformation, not only behavioral compliance (Verlind-Brouwer et al., 2025; R. Cruess et al., 2016).

Complementing this view, Jill Goldie describes identity as a dynamic process shaped through interaction with the learning environment. Medical education therefore reshapes how students perceive themselves and their place within the profession. In this process, identity capital—personal resources such as socioeconomic context, gender, self-esteem, or critical thinking—can either facilitate or hinder professional development. Conflicts between institutional and personal values may generate identity dissonance, which has been associated with stress and potential attrition from training programs (Goldie, 2012). Similarly, David M. Irby and Sanne Hamstra distinguish virtue, behavioral, and identity approaches to professionalism, with the latter emphasizing the integration of professional values into the student’s self-concept (Sitnik et al., 2023).

Kalet and colleagues emphasize that PIF does not follow a linear or purely cumulative trajectory. Instead, it involves advances, tensions, and setbacks over time, a dynamic also highlighted by Goldie in her research. This evolving nature underscores the importance of longitudinal or repeated quantitative measurement to capture these fluctuations in identity development (Goldie, 2012; Kalet et al., 2018).

Cooperative behavior changes according to the structure of interdependence; that is, how one person’s actions influence their own and others’ outcomes (S. R. Cruess et al., 2019). Various educational processes have been observed that promote the construction of professional identity during the training stage. These include spontaneous reflection on experiences in both academic and professional settings; reflection guided by teachers and tutors; formative feedback; observation of professional performance models; and open dialogue on conceptual aspects related to personal and professional identity, as well as on the experiences that arise from each student’s identification process (Balliet & Lindström, 2023).

In health education, supporting the basic psychological needs proposed by Self-Determination Theory promotes the development of professional identity. In particular, autonomy support from mentors and training environments influences important professional decisions, such as specialty choice, and encourages more active engagement in clinical learning (Ryan & Deci, 2020). A recent meta-analysis by Nikolaos Ntoumanis found that supporting autonomy, competence, and relatedness was associated with significant improvements in health behaviors and psychological well-being (Ntoumanis et al., 2021). Despite the availability of these robust frameworks, an important knowledge gap remains: there is a lack of quantitative evidence that directly connects collaborative interdependence with norms and belonging and, ultimately, with professional identity formation in preclinical cohorts within collaborative learning contexts such as PBL or TBL.

Therefore, the objective of this article was to examine the associations between perceived social interdependence in collaborative learning activities and professional identity among fourth-semester medical students enrolled in a Musculoskeletal and Integumentary System course during the 2026 academic cycle.

## 2. Materials and Methods

This study employed a quantitative, cross-sectional, correlational, non-experimental design with a non-probabilistic convenience sampling strategy, including 108 fourth-semester medical students from the School of Medicine who voluntarily agreed to participate.

In the academic program of the Musculoskeletal and Integumentary System course, offered during the fourth semester at the School of Medicine of the Universidad Autónoma de Guadalajara (Zapopan, 45129, Mexico), group-based activities function as the integrative axis of the lecture sessions. The purpose of these collaborative activities is to promote supervised self-directed learning in real time and in person, under the direct guidance of the instructor. Within these sessions, each student engages in teamwork to develop clinical scenarios, participate in structured discussion panels, and deliver research presentations. In the clinical scenario activity, students receive a clinical note accompanied by laboratory and imaging results; each team must propose a primary and a secondary diagnosis, justified with reference to clinical criteria and both national and international guidelines. They must also explain, from a pathophysiological standpoint, the relevance of each variable associated with the diagnosis, justify expected laboratory and imaging findings to confirm it, and finally present an evidence-based schematic or syntax aligned with the current medical guideline for the condition. For the discussion panel activity, each team selects and answers a research objective related to a clinical topic and conducts an expert-style academic session in which they defend their arguments with referenced and well-substantiated evidence. Finally, in the research presentation, the team delivers a plenary exposition covering the anatomy, physiology, pathophysiology, and clinical analysis of a pathology designated by the instructor.

### 2.1. Didactic Procedure

To address the objective of this study—to examine the relationship between social interdependence in collaborative activities and the development of professional identity among fourth-semester medical students enrolled in a Musculoskeletal and Integumentary System course during the 2026 academic cycle, and in accordance with the course’s curriculum—ten clinical cases were implemented over the semester. These cases covered lower-extremity pathology, upper-extremity pathology, posture and gait disorders, vertebral column pathology, extracellular matrix disorders, growth disorders, rheumatic conditions, neuromuscular junction pathology, inflammatory myopathies, and toxic myopathies. Additionally, each team completed one oral presentation and one structured panel discussion. The topics for these activities varied depending on the instructional unit being covered at the time (muscular anatomy, integumentary system, bone tissue, cartilage, or muscle tissue).

### 2.2. Sample and Design

Participants who met the following inclusion criteria were enrolled: (1) medical students; (2) enrollment at the Universidad Autónoma de Guadalajara; (3) age ≥ 18 years; (4) voluntary participation.

### 2.3. Variables

The study variables were defined as follows: the dependent variable—medical professional identity (PIF attachment and PIF detachment); and the independent variables—collaborative work (outcome interdependence, means interdependence, and boundary interdependence). These variables were examined using two validated instruments. The Professional Identity Questionnaire (Toben et al., 2021) measures levels of attachment and identification with the medical professional group, as well as attitudes of distancing or detachment, allowing analysis of the formative process through which students internalize values, behaviors, attitudes, belonging, and self-concept as future physicians. This instrument consists of 10 items. The Social Interdependence in Collaborative Learning Scale (Shimizu et al., 2020) measures perceived positive interdependence within collaborative activities in health science educational contexts, allowing analysis of the process through which students work together to construct knowledge through positive social interdependence, exchange of ideas, joint problem solving, and mutual support within group-based activities. This instrument consists of 15 items.

### 2.4. Instruments

To evaluate collaborative learning and professional identity development, two validated instruments were used. The Social Interdependence in Collaborative Learning Scale (SOCS) measures perceived interdependence. Professional identity was assessed using the Professional Identity Questionnaire (PIQ). In addition, it shows concurrent validity by correlating with autonomous motivation as described by Self-Determination Theory, making it useful for evaluating the impact of the curriculum on professional development (Shimizu et al., 2020; Ryan & Deci, 2020; Ntoumanis et al., 2021; Lv et al., 2025).

#### 2.4.1. Social Interdependence in Collaborative Learning Scale (SOCS)

The SOCS was developed to provide a rigorous and theoretically grounded instrument capable of measuring social interdependence within collaborative learning environments. Its construction followed a modified Delphi process involving faculty members, educational experts, and a sample of 264 students from various health professions participating in a structured collaborative learning activity. These students, enrolled in programs such as medicine, nursing, physiotherapy, occupational therapy, and medical technology, worked in groups. The initial stage consisted of an extensive literature review that identified eighty-six items related to interdependence, cooperation, and collaboration across educational settings. This preliminary list was subsequently refined to thirty-seven items through a conceptual review aimed at reducing redundancy and improving clarity. The Delphi panel comprised three distinct groups—medical students in their clinical years, education specialists, and medical educators with postgraduate training in teaching and experience in collaborative learning—representing eight countries. After two rounds of consensus, a sixteen-item version was established, although one item was later removed due to a correlation exceeding 0.70 with another, indicating redundancy.

Confirmatory factor analysis revealed that the SOCS exhibits an excellent three-factor structure, with strong indices of model fit, including a Goodness-of-fit index (GFI) of 0.924, a Comparative Fit Index (CFI) of 0.951, a Mean squared error of approximation (RMSEA) of 0.061, and a CMIN/df of 1.838. Each of the three dimensions—outcome interdependence, means interdependence, and boundary interdependence—demonstrated high internal consistency, with Cronbach’s alpha coefficients of 0.818, 0.866, and 0.811 respectively. The three SOCS dimensions illuminate essential processes in collaborative learning: outcome interdependence reflects the group’s commitment to shared goals and mutual academic support; means interdependence concerns the coordination of resources, roles, and reciprocal assistance that sustain joint work; and boundary interdependence encompasses group identity, cohesion, interpersonal respect, and the integration of diverse viewpoints. Together, these dimensions provide a comprehensive assessment of the interpersonal dynamics that underpin collaborative learning. As a result, the SOCS emerges as a robust and relevant instrument for examining social interdependence in higher education and professional training contexts. Its development and validation contribute significantly to the scholarly understanding of how students co-construct knowledge and how social interdependence shapes collaborative learning processes (Shimizu et al., 2020).

#### 2.4.2. Professional Identity Questionnaire (PIQ)

The Professional Identity Questionnaire functions as a quantitative and theoretically grounded instrument for assessing professional identity formation (PIF) within medical education. Its implementation allows educators to monitor how the formal, informal, and hidden curriculum shapes students’ identification with the medical profession, offering a systematic alternative to time-intensive qualitative methods. In parallel, experiential learning opportunities and structured contact with practicing professionals reinforce students’ competence, autonomy, and sense of relatedness—elements widely recognized as drivers of professional identity formation. Given that PIF reflects an internalization process through which values and professional roles are adopted, educational environments must be intentionally designed to support basic psychological needs. Monitoring both positive (attachment) and negative (detachment) attitudes toward the profession enables instructors to detect unintended curricular effects and adjust pedagogical approaches accordingly. The PIQ has demonstrated applicability among medical students, providing a numerical representation of one’s sense of belonging to the medical profession. The instrument comprises ten items rated on a five-point Likert scale, where items A–E are positively worded and items F–J are negatively worded, requiring reverse scoring before summation. Factor analysis in medical student populations identifies a two-factor model: professional identity attachment (PIF attachment), reflecting pride, belonging, and emotional connection, and professional identity detachment (PIF detachment), indicating discomfort, rejection, or the impulse to justify one’s membership in the group. Measurement invariance across gender confirms that men and women interpret the instrument in comparable ways, supporting unbiased comparisons and strengthening construct validity.

Internal consistency is high, with a Cronbach’s alpha of 0.82, exceeding thresholds commonly recommended for applied research. Confirmatory factor analysis produces indices indicating good to excellent model fit, including CFI values ranging between 0.945 and 0.981, TLI values between 0.929 and 0.973, and RMSEA values between 0.053 and 0.080. Concurrent validity is evidenced by the positive associations between PIQ scores and autonomous forms of motivation (r ≈ 0.35–0.37), coupled with a negative association with external regulation (r ≈ −0.13), aligning with Self-Determination Theory and reinforcing the conceptual link between PIF and motivational internalization. Expert evaluation using item-level content validity indices (I CVI) further supports the instrument’s relevance, although only four items (B, C, D, and E) were unanimously identified as highly pertinent.

Interpretation of the total PIQ score provides insight into the strength of the student’s professional identity. Higher scores reflect stronger attachment—manifested as pride, belonging, and identification—and lower levels of detachment. These scores correlate with more autonomous forms of motivation, indicating that students with high PIQ scores endorse professional values more fully and engage in their training with greater internal commitment. Regular measurement thus aids in identifying curricular components that inadvertently promote detachment and in guiding targeted interventions that strengthen belonging and internalization. The PIQ assesses social identification with the medical profession as a collective reference group, rather than evaluating the doctor–patient relationship or the student–teacher dynamic. Several items explicitly probe pride in belonging, the strength of ties, and the emotional sense of identification, all of which align with the developmental trajectory of “becoming” a physician (Toben et al., 2021).

### 2.5. Language Adaptation

The Professional Identity Questionnaire (PIQ) and the Social Interdependence in Collaborative Learning Scale (SOCS) were originally developed and validated in English. For the purposes of this study, both instruments were translated into Spanish by the authors, given the absence of a previously validated Spanish version. The translation focused on semantic equivalence and conceptual clarity to ensure comprehension by Mexican medical students. The instruments were used for exploratory analytical purposes. The internal consistency of the translated questionnaire was assessed using Cronbach’s alpha. The instrument demonstrated adequate internal consistency, with a Cronbach’s alpha of 0.791 for the total questionnaire. Items related to the attachment subscale had a Cronbach’s alpha of 0.826, and those in the detachment questionnaire had a Cronbach’s alpha of 0.950.

No factor analysis was conducted in the present study, as the instrument was used for exploratory purposes and based on previously validated structures reported in the literature.

### 2.6. Ethical Principles

The present study was conducted in accordance with the Helsinki declaration, ensuring the confidentiality of personal data by assigning an identification consecutive alphanumeric code to each patient (TNI XXX). Furthermore, only the researchers involved in the study had access to the data obtained during the study. We obtained informed consent from all subjects included in the study.

### 2.7. Statistical Analysis

Statistical analyses were conducted to explore associations between perceived social interdependence and professional identity. Descriptive statistics were used to summarize the data: continuous variables were expressed as means and standard deviations, while categorical variables were presented as frequencies and percentages. For comparative analyses between students with a medium versus high sense of professional belonging, chi-square (χ^2^) tests were applied to categorical variables, and Student’s *t*-tests for independent samples were used to compare continuous variables. Associations between professional identity scores (attachment and detachment) and the dimensions of social interdependence were examined using Pearson’s correlation coefficients. In addition, multiple linear regression analyses were planned to examine the extent to which dimensions of social interdependence jointly accounted for variability in professional identity scores, while allowing estimation of independent associations within an exploratory framework. All analyses were conducted for exploratory purposes, in accordance with the correlational nature of the study. A statistical significance level of *p* ≤ 0.05 was adopted, corresponding to a 95% confidence level. No causal or predictive inferences were drawn from the statistical analyses. Statistical analyses were performed using IBM SPSS Statistics, version 26 (IBM Corp., Armonk, NY, USA).

## 3. Results

In Table 1 we show the sociodemographic variables of the total number of participants. It can be observed that more than 60% of the population was female. Nearly 90% were between 19 and 20 years old, more than 80% chose medicine as their first-choice major, and 75% already had experience in health-related activities.

In Table 2, we describe the elements of comparison between the construct of professional identity as healthcare personnel among university students. It was observed that 86% of the population identified as part of the medical profession. Furthermore, there is a high level of attachment to medicine, over 90%, as well as strong peer relationships for the development of collaborative activities; notably, more than 90% of the subjects are interested in collaborative learning.

Table 3 presents the comparison between medium and high levels of sense of belonging. We identified the following factors associated with a higher sense of belonging: an age between 19 and 20 years old (73.3% vs. 92.5%, *p* = 0.04), having medicine as the first-choice major (60% vs. 86%, *p* = 0.02), and lower percentage of parents working within the medical field (60% vs. 29%, *p* = 0.02).

Table 4 presents the correlations between the overall professional identity score and the variables of interest. A very strong positive correlation was observed with the professional identity detachment score (*p* < 0.001), as well as a strong positive correlation with professional identity attachment (*p* < 0.001). Moderate correlations were identified with the perceived importance of team-based learning and peer collaborative work (*p* < 0.001), while lower correlations were observed with perceived limitations related to collaborative work resources (*p* < 0.001). Items assessing professional identity detachment (items F–J) were reverse-scored prior to analysis, such that higher detachment scores represented lower levels of professional distancing.

In Table 5, the multivariable analysis, the first model shows that a positive association was observed between the total collaborative work score and the sense of identification (B = 0.248, β = 0.361, 95% CI: 0.129 to 0.367, *p* < 0.001). This was similar for the first career choice variable, which showed a significant negative association with the identification score (B = −3.711, β = −0.258, 95% CI: −6.122 to −1.300, *p* = 0.003). In the forward stepwise method, these two factors retained their status as independent associated factors.

In this multiple linear regression model, the variables initially considered were age 19–20 years, medicine as first career choice, parents working as healthcare personnel, and total collaborative work score. After the stepwise selection procedure, only total collaborative work score and medicine as first career choice remained in the final model. In Step 1, total collaborative work score was entered into the model and explained 17.4% of the variance in the dependent variable, as indicated by an R^2^ change of 0.174. In Step 2, the inclusion of medicine as first career choice produced an additional R^2^ change of 0.061, indicating that this variable explained an additional 6.1% of the variance beyond that already explained by total collaborative work score.

## 4. Discussion

The findings of this study support the relevance of Social Interdependence Theory (SIT) within collaborative learning contexts in preclinical medical education. The results suggest that social interdependence should be understood as a multidimensional construct encompassing boundaries, team learning, and shared resources, rather than as a single, undifferentiated phenomenon. In particular, the dimension related to group boundaries highlights the importance of perceived cohesion, shared identity, and mutual respect within learning communities, reinforcing the idea that collaborative learning is sustained through clearly defined group structures and social ties (Wittek et al., 2025; Shimizu et al., 2022).

From a conceptual standpoint, boundary interdependence emphasizes how groups maintain internal cohesion while simultaneously defining distinctions from other groups. This perspective aligns with prior research indicating that collaborative learning environments benefit from strategies that promote coordination of opinions, reciprocal respect, and structured exchange of tasks and resources. In this regard, the present findings are consistent with previous studies showing that cooperative strategies such as task integration, debate, and structured interaction foster collaborative work (Peralta Ortega et al., 2025).

Similarly, the dimensions related to shared resources and team learning underscore the dual contribution of cognitive and social components to effective collaboration. While confidence in shared information supports technical aspects of group work, team learning reflects an attitudinal orientation toward mutual support and collective engagement. This convergence is consistent with earlier reports emphasizing that successful collaborative learning depends on both cognitive competence and the quality of social interactions among learners (Torres et al., 2025; Castro et al., 2021).

Likewise, a strong association was observed between sense of belonging (PIF attachment) and perceived social interdependence. From a theoretical perspective, this relationship may reflect how mutually supportive learning environments are conceptually linked to students’ professional identification. Social Interdependence Theory and Social Identity Theory suggest that positive interdependence can provide a contextual setting that supports cooperation, shared goals, and group cohesion (Johnson & Johnson, 2009; Whelan, 2025). However, in the present study, these relationships were examined exclusively at an associative level. No causal pathways or mediational mechanisms were empirically tested, and interpretations should therefore be limited to theoretical explanations consistent with the observed correlations. Rodríguez Castillo and Figueroa Coronado (2022) demonstrated a direct relationship between collaborative work and the strengthening of interpersonal skills. Fergusson et al. (2021) and Zhang and Chen (2023) reaffirm that integrating cooperative work with practice-based learning models amplifies the benefits of situated learning, generating a stronger professional identity (Rodríguez Castillo & Figueroa Coronado, 2022; Fergusson et al., 2021; Zhang & Chen, 2023).

The characteristics of the study population also provide relevant contextual considerations. A substantial proportion of participants reported prior experience in health-related settings, which may contribute to the early consolidation of social and professional skills through observational learning and behavioral modeling within family or community contexts. Additionally, the predominance of participants from rural environments raises the possibility that contextual background influences the nature and frequency of social interactions that shape collaborative competencies. Previous research suggests that the development of social skills relies heavily on experiential learning and interpersonal contact, and that limitations in social interaction may affect broader academic and professional performance (Mulyana et al., 2024).

Statistical analyses indicated a strong association between professional identification and attachment dimensions. In this sample, a high proportion of participants (82%) reported having chosen medicine as their first vocational option, a characteristic that was associated with higher levels of professional identity attachment (r = 0.725, *p* < 0.001). This pattern suggests that early vocational commitment co-occurs with stronger affective identification with the medical profession. Similarly, the strong association observed between professional identity attachment and detachment (r = 0.877, *p* < 0.001) indicates a close inverse relationship between these two dimensions within the professional identity construct. Higher levels of professional identity coherence were associated with lower levels of detachment, while weaker or more fragmented identification with the medical profession was associated with higher detachment scores. These findings reflect patterns of association between identity-related attitudes rather than directional or developmental processes, given the cross-sectional and correlational nature of the study. Conversely, a weak or fragmented group identity can lead to higher levels of detachment, which could compromise the physician’s integration into their community of experts (Toben et al., 2021).

Another important aspect is the demographic distribution, which is consistent with 90% of students being between 19 and 20 years old, and 61% being women. This trend is explained by what Kwiek and Roszka (2021) describe, suggesting that there are significant disparities in cooperative modalities according to gender. It is observed that the female sector shows a preference for structured collaboration schemes, characterized by the definition of clear processes and a systematic organization of group tasks (Kwiek & Roszka, 2021).

Social Interdependence Theory (SIT) highlights that social norms are fundamental to structuring group interactions, as they regulate behaviors and expectations that facilitate positive cooperation and the achievement of common goals (Butera & Buchs, 2019; Rusbult & van Lange, 2003). Social cognition plays a key role in how individuals perceive and respond to interdependence, interpreting cues and adjusting their behaviors to maintain effective cooperative relationships (Balliet & Lindström, 2023; Rusbult & van Lange, 2003). Social interdependence implies that individual outcomes depend on the actions of others, which fosters cooperative behavior when shared goals and clear mutual responsibilities are established (Johnson, 2003; Johnson & Johnson, 2005; Butera & Buchs, 2019). In professional contexts, this cooperation influences the construction of professional identity, integrating new attitudes, values, and practices within a community of practice, which strengthens the sense of belonging and commitment to the group (Johnson & Johnson, 2017; Shimizu et al., 2022). Furthermore, the interaction between individual and social agency is relationally interdependent, where professional identity is shaped by both personal experiences and social influences accumulated over time (Billett, 2006). Taken together, these elements show social norms, cognitive processes, and structures of interdependence. They shape cooperative behavior and the formation of professional identities in collaborative environments (Balliet et al., 2017; Lange & Balliet, 2012).

The findings of this study demonstrate that boundary interdependence is the strongest correlate of collaborative work, that may indicate that collaboration tends to be higher in contexts where group cohesion and shared identity are present. Mere goal alignment is insufficient; it is essential that students perceive themselves as part of a team. Although Social Interdependence Theory suggests that goal interdependence may initiate cooperation, our results indicate that, in the preclinical stage, belonging to a stable group is key to sustaining interaction over time (Johnson & Johnson, 2009; Shimizu et al., 2020).

The multiple linear regression analyses indicate that perceived collaborative work shows a consistent and independent association with professional identification among preclinical medical students. Notably, this relationship remained stable after controlling for key sociodemographic and vocational variables, including age, selection of medicine as a first-choice career, and parental involvement in the medical profession. The persistence of this association across analytical approaches, and its increased explanatory contribution in the reduced model, suggests that collaborative learning experiences capture variance in professional identification beyond individual background characteristics.

Although the cross-sectional design of the study precludes causal interpretation, these findings highlight collaborative learning environments as a salient contextual factor associated with students’ sense of belonging and identification with the medical profession. The robustness of the association across models underscores the potential relevance of intentionally structured collaborative work during the preclinical stage, when professional identity is still emerging. From an educational perspective, this pattern supports the consideration of collaborative learning structures as part of curricular designs aimed at fostering students’ early engagement, identification, and integration into the professional community of medicine.

This is consistent with professional identity formation as a process of socialization based on belonging and interaction within a community of practice (R. Cruess et al., 2016; Goldie, 2012). In this regard, at the preclinical level of medical education, educational design should move beyond simply organizing students into groups and instead structure structured collaborative learning approaches such as problem-based learning or team-based learning sessions that ensure sustained interaction throughout the course. This organization may be particularly relevant for students with diverse sociodemographic backgrounds or varying levels of prior exposure to health-related environments, for whom predictable structures and clearly defined roles can provide additional support during collaborative activities. Such approaches can be implemented through fixed teams maintained over time, combined with the assignment of rotating functional roles within each session (e.g., information seeker, evidence synthesizer), allowing work to be organized in an explicitly interdependent manner. In addition, clinically oriented case-based tasks and increasing complexity should be used to require integration of contributions across all team members rather than isolated activities. In this structure, each student’s performance becomes directly linked to the collective output of the team, reinforcing mutual accountability and supporting the internalization of shared professional norms aligned with medical practice.

Given the dynamic nature of professional identity formation, the effectiveness of these strategies should be evaluated using longitudinal measurements. Validated instruments such as the SOCS and PIQ are recommended at three points during the semester (beginning, mid-course, and end) to monitor changes in perceived interdependence and professional identity. This follow-up allows for the timely identification of professional disengagement and supports evidence-based pedagogical adjustments, promoting identity development prior to the transition into clinical settings (Kalet et al., 2018; Ryan & Deci, 2020).

The limitations of this study should be acknowledged. First, the sample was limited to a non-probabilistic convenience sample of 108 fourth-semester medical students from a single medical school and a specific course within one academic institution. As a result, the findings may not be generalizable to medical students in other academic years, institutions, or educational contexts. The results should therefore be interpreted as context-specific and exploratory in nature. Future studies incorporating larger, probabilistic, and multi-institutional samples are needed to enhance external validity and to examine whether the observed associations hold across diverse medical education settings. Nevertheless, a limitation of this study is the use of Spanish translations of the PIQ and SOCS that have not yet undergone formal cross-cultural validation. The instruments were used for exploratory analytical purposes, and measurement equivalence cannot be fully assured. The results should therefore be interpreted as preliminary and context-specific. Validated instruments such as the SOCS and PIQ are recommended at three points during the semester (beginning, mid-course, and end) to monitor changes in perceived interdependence and professional identity. This follow-up allows for the timely identification of professional disengagement and supports evidence-based pedagogical adjustments, promoting identity development prior to the transition into clinical settings.

Future research should adopt longitudinal study designs with larger and more diverse samples, including students from multiple academic years, medical programs, and universities. Expanding the population and institutional scope would enhance external validity and allow examination of how perceived social interdependence and professional identity evolve across different stages and contexts of medical education. Given the dynamic nature of professional identity formation, the effectiveness of these strategies should be evaluated using longitudinal measurements.

## 5. Conclusions

This study examined the association between perceived social interdependence in collaborative learning activities and professional identity among preclinical medical students. The findings indicate that collaborative work is consistently and independently associated with higher levels of professional identification, even after controlling for relevant sociodemographic and vocational factors. In particular, dimensions related to group cohesion, team boundaries, and coordinated use of shared resources were closely linked to students’ sense of belonging to the medical profession.

Although the cross-sectional design precludes causal inferences or conclusions about developmental change over time, the results suggest that collaborative learning environments constitute an important educational context in which social interdependence and professional identity-related attitudes co-occur. From an educational perspective, these findings support the intentional design of structured collaborative activities during the preclinical stage, when professional identity is still emerging.

Overall, this study provides exploratory quantitative evidence connecting social interdependence and professional identity in early medical education, integrating theoretical perspectives from Social Interdependence Theory and professional identity formation frameworks with empirical data. Future research should employ longitudinal, multi-institutional designs and more advanced analytical approaches to further examine the dynamic processes and mechanisms underlying professional identity formation across the medical curriculum.

## Figures and Tables

**Table 1:** Sociodemographic variables of the subjects.

Variable	Population *n* = 108
Female	66 (61.1)
Age	
- 17–18	1 (0.9)
- 19–20	97 (89.8)
- 21–22	10 (9.3)
Medicine as first-choice major	89 (82.4)
Parents’ education level	
- Primary school	1 (0.9)
- Middle school	11 (10.2)
- High school	6 (5.6)
- Bachelor’s degree	54 (50)
- Postgraduate degree	36 (33.3)
Urban area of residence	14 (13)
Parents’ profession related to healthcare	36 (33.3)
Experience in healthcare activities	82 (75.9)

Qualitative variables are expressed as frequencies and percentages.

**Table 2:** Description of the elements of comparison for the healthcare professional identity construct.

Variable	Population *n* = 108
Professional attachment score	22.18 ± 2.77
Professional attachment classification	
- Low attachment	1 (0.9)
- Medium attachment	7 (6.5)
- High attachment	100 (92.6)
Professional detachment score	20.31 ± 3.97
Professional detachment classification	
- Low detachment	81 (75)
- Medium detachment	22 (20.4)
- High detachment	5 (4.6)
General medical identification score	42.49 ± 5.50
Medical identification classification	
- Medium sense of belonging	15 (13.9)
- High sense of belonging	93 (86.1)
Boundaries score	36.53 ± 5.17
Boundaries classification	
- Low	1 (0.9)
- Medium	20 (18.5)
- High	87 (80.6)
Team-based learning score	13.63 ± 1.90
Team-based learning classification	
- Low importance	1 (0.9)
- Medium importance	8 (7.4)
- High importance	99 (91.7)
Means/Resources score	12.49 ± 2.13
Means/Resources classification	
- Low	2 (1.9)
- Medium	27 (25)
- High	79 (73.1)
Collaborative work score	62.65 ± 7.99
Collaborative work classification	
- Low	1 (0.9)
- Medium	13 (12)
- High	94 (87)
Type of collaborative work	
- Clinical case	61 (56.5)
- Presentation	30 (27.8)
- Panel	15 (13.9)
- Other	2 (1.9)

Qualitative variables are expressed as frequencies and percentages; quantitative variables are expressed as means and standard deviations.

**Table 3:** Comparison of sociodemographic and academic factors in subjects with medium vs. high sense of identification as healthcare personnel.

Variable	Medium Sense of Belonging *n* = 15	High Sense of Belonging *n* = 93	*p*
Female	8 (53.3)	58 (62.4)	0.50
Age 19–20 years	11 (73.3)	86 (92.5)	0.04
First career choice	9 (60)	80 (86)	0.02
Parents’ education level (bachelor’s and postgraduate)	13 (86.7)	77 (82.8)	0.76
Rural origin	14 (93.3)	80 (86)	0.69
Parents as healthcare personnel	9 (60)	27 (29)	0.02
Experience in healthcare activities	14 (93.3)	68 (73.1)	0.11
High acceptance of collaborative work	12 (80)	82 (88.2)	0.15
Type of collaborative work			
- Clinical case	6 (40)	55 (59.1)	
- Presentation	4 (26.7)	26 (28)	
- Panel	5 (33)	10 (10.8)	0.12
- Other	0 (0)	2 (0)	

Quantitative variables are expressed as means and standard deviations; qualitative variables as frequencies and percentages. Comparison between groups: chi-squared test for qualitative variables and Student’s *t*-test for quantitative variables. Statistical significance: *p* ≤ 0.05.

**Table 4:** Correlation between healthcare professional identity score and variables of interest.

Variable	Total Healthcare Professional Identity Score
	r
Total attachment score	0.725 **
Total detachment score	0.877 **
Total boundaries score	0.329 **
Total team-based learning score	0.420 **
Total means/resources score	0.392 **
Total collaborative work score	0.417 **

Pearson correlation. Statistical significance: * *p* ≤ 0.05, ** *p* ≤ 0.01.

**Table 5:** Multiple linear regression models for factors associated with a sense of identification as healthcare personnel.

	Intro Method				Backward Stepwise Method			
*n* = 107	B	β	CI 95%	*p*	B	β	CI 95%	*p*
Age 19–20 years	1.893	0.105	−1.227–5.013	0.232				
First career choice	−3.711	−0.258	−6.122–−1.3	0.003	−3.54	−0.246	−5.974–−1.105	0.005
Parents as healthcare personnel	1.711	0.147	−0.276–3.697	0.091				
Total collaborative work score	0.248	0.361	0.129–0.367	<0.001	0.281	0.409	0.165–0.398	<0.001

A multivariate linear regression analysis was performed using the Enter and Stepwise methods, with results presented as B coefficients, β coefficients and 95% confidence intervals.

## Data Availability

The datasets generated and analyzed during the current study are available from the corresponding author upon reasonable request.

## Abbreviations

The following abbreviations are used in this manuscript:

SIT	Social Interdependence Theory
SOCS	Social Interdependence in Collaborative Learning
PIQ	Professional Identity Questionnaire
PIF	Professional Identity Formation
